# Referral and adjuvant treatment patterns after nephrectomy in high‐risk locoregional renal cell carcinoma

**DOI:** 10.1002/cam4.4407

**Published:** 2021-11-09

**Authors:** Hannah Dzimitrowicz, Elizabeth Esterberg, LaStella Miles, Giovanni Zanotti, Azah Borham, Michael R. Harrison

**Affiliations:** ^1^ Division of Medical Oncology Department of Medicine Duke Cancer Institute Duke University Durham North Carolina USA; ^2^ RTI Health Solutions Research Triangle Park North Carolina USA; ^3^ Pfizer Inc New York New York USA

**Keywords:** adjuvant therapy, kidney cancer, sunitinib

## Abstract

**Background:**

It is unclear whether patients with renal cell carcinoma (RCC) are routinely assessed for recurrence risk post‐nephrectomy and whether patients at high recurrence risk are seen by providers who can evaluate candidacy for adjuvant systemic therapy (AST) and clinical trials.

**Materials and Methods:**

We identified all patients with locoregional RCC who underwent nephrectomy via an institutional database within Duke University Health System between 1 April 2015 and 31 December 2019. Medical records were reviewed to identify patient characteristics, post‐nephrectomy referrals, treatment, and follow‐up. Patients with tumor stage ≥3 and grade ≥2, regional lymph node metastasis, or both, were classified as high recurrence risk.

**Results:**

Of 618 patients with locoregional RCC who underwent nephrectomy, 136 (22%) had high recurrence risk. Of those, 25 patients with high‐risk disease (18%) were referred to medical oncology for discussion of AST; 23 (92%) of these referrals took place in 2018–2019. One patient received adjuvant sunitinib and two patients participated in adjuvant immunotherapy trials. The decision not to receive AST was primarily made by the oncologist in 10 (46%), the patient in 8 (36%), and unrecorded in 4 (18%) of 22 cases, for multiple reasons. Individual surgeons referred high‐risk patients for discussion of AST with varying frequency, ranging from 0% to 100% in 2019.

**Conclusions:**

Despite increasing number of patients with locoregional RCC at high recurrence risk referred to medical oncologists after nephrectomy, few patients received AST, including participation in clinical trials. With increasing AST options and ongoing clinical trials in this space, these findings highlight the need for continued efforts at identifying effective AST and referring patients most likely to benefit to medical oncologists.

**ClinicalTrials.gov,** NCT04309617.

## Graphical abstract

We identified all patients with locoregional renal cell carcinoma who underwent nephrectomy between 1 April 2015 and 31 December 2019 to characterize patient characteristics, patients at high recurrence risk, post‐nephrectomy referrals, treatment, and follow‐up. Despite increasing number of patients with locoregional RCC at high recurrence risk referred to medical oncologists after nephrectomy (23 of 25 referrals taking place in 2018‐2019), few patients (n=3) received adjuvant systemic therapy, including participation in clinical trials.

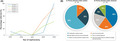

## 1 INTRODUCTION

In the United States, renal cell carcinoma (RCC) accounts for approximately 4% of new cancer cases and will result in an estimated 13,780 deaths in 2021.^1^ Five‐year survival for patients with RCC is 75%^2^; however, prognosis varies by disease stage. Five‐year survival rates for patients diagnosed with regional and metastatic disease are 70% and 13%, respectively.^2^

The primary treatment for patients with localized or regional disease is nephrectomy with curative intent followed by surveillance.^3^ Despite this intervention, up to 40% of patients initially diagnosed with locoregional disease will experience subsequent recurrence and distant metastases.^4^, ^5^ The risk of recurrence, however, is not equal for all patients and efforts have been made to identify patients at highest risk of recurrence and subsequent mortality, resulting in several published risk scoring systems. Prominent among these risk predictors is the UCLA Integrated Staging System (UISS), which incorporates three factors: TNM (tumor, node, and metastasis stage), Eastern Cooperative Oncology Group performance status (ECOG PS), and Fuhrman nuclear grade. These factors can classify patients with RCC as low, intermediate, or high risk with 5‐year survival rates of 92%, 67%, and 44%, respectively.^6^, ^7^ Patients at highest risk of recurrence carry the greatest need for additional interventions to prevent or delay recurrence in order to improve survival.

Given the risk of recurrence and subsequent mortality, there have been numerous and ongoing efforts to identify effective adjuvant systemic therapy (AST), although its role in RCC remains controversial. On 16 November 2017, sunitinib was approved by the US Food and Drug Administration (FDA) for the adjuvant treatment of patients at high risk of recurrent RCC after nephrectomy, making it the first approved AST for RCC. This approval was based on the results of the phase III S‐TRAC trial in which patients treated with adjuvant sunitinib for 12 months after nephrectomy had improved disease‐free survival compared with patients who received placebo (6.8 vs. 5.6 years; hazard ratio 0.76; 95% confidence interval [CI], 0.59–0.98; *p* = 0.03); however, an overall survival (OS) benefit was not demonstrated.^8^, ^9^ In contrast, multiple other trials of AST in RCC, including tyrosine kinase inhibitors that have demonstrated benefit in the treatment of metastatic RCC, have failed to demonstrate benefit in the adjuvant setting.^10^, ^11^, ^12^, ^13^ There are multiple ongoing trials evaluating alternative adjuvant therapies, including checkpoint inhibitor immunotherapy, many with pending results. The first of these adjuvant immunotherapy studies with available results, KEYNOTE‐564, demonstrated improved disease‐free survival with adjuvant pembrolizumab compared to placebo in patients with intermediate‐high, high risk, or M1 NED (no evidence of disease after resection) RCC.^14^

Despite the potential role that treatment with adjuvant sunitinib and trials of alternative AST may play in the treatment of patients with resected RCC, it is unclear whether patients with RCC are routinely assessed for recurrence risk post‐operatively, whether patients at high risk of recurrence are seen by medical oncologists who can evaluate candidacy for AST and clinical trials, and what proportion of patients in real‐world practice are receiving adjuvant therapy. To address these questions, we performed a retrospective chart review of all patients with locoregional RCC who underwent nephrectomy within Duke University Health System (DUHS) to characterize referral and adjuvant treatment patterns after nephrectomy.

## 2 MATERIALS AND METHODS

### 2.1 Patient population

We identified all patients with locoregional RCC (no distant metastases at time of diagnosis) who underwent nephrectomy via an institutional database within DUHS between 1 April 2015 and 31 December 2019. Patients aged 18 years or older at the time of nephrectomy were eligible. This study was approved by the institutional review board and waivers for obtaining informed consent were granted.

### 2.2 Data collection

The electronic medical record of each patient with locoregional RCC who underwent nephrectomy within DUHS was reviewed manually. The following data items were collected for all eligible patients when available: patient demographics, including age, race, and ECOG PS; tumor characteristics, including date of diagnosis, tumor and nodal stages, tumor grade, and clear cell predominance; treatment characteristics, including type of nephrectomy (full or partial); time to post‐operative follow‐up; and follow‐up data, including plan at first follow‐up appointment, any subsequent recurrence, and last follow‐up date.

Using a modified version of the UISS, patients were classified as being modified high risk of recurrence if RCC was tumor stage 3 or higher combined with a tumor grade 2 or higher, regional lymph node metastasis, or both. UISS high‐risk disease was defined as T3, grade ≥2, and ECOG ≥1; T4; or N1 disease.^6^ ECOG PS was available for only 44% of patients in this study, limiting its use in our study. Full UISS risk was also recorded when ECOG PS was available.

For patients at modified high risk of recurrence, additional data regarding post‐nephrectomy referral and treatment decisions were collected, including referrals for RCC‐related care, whether patients received AST and, if so, details of treatment type and duration, recorded reasons for decisions not to receive AST, and participation in clinical trials.

### 2.3 Statistical analysis

All analyses were descriptive and conducted using SAS version 9.4. Variables were summarized descriptively through the tabular and graphical display of total (n) and percentage (%), mean values, 95% CI, median, quartile, and standard deviation (SD) or standard errors for continuous variables of interest and frequency distributions for categorical variables. All eligible patients were included in descriptive analyses of variables pertaining to patient characteristics, baseline clinical characteristics, and initial post‐operative follow‐up visits. Patients who were categorized as having modified high risk of recurrence had additional descriptive analyses performed, including referral patterns, treatment patterns, and health outcomes, as well as time‐to‐event measures (recurrence‐free survival and OS). To adjust time‐to‐event measures, a Kaplan–Meier method that accounts for right censoring was implemented.^15^ To evaluate time to recurrence, the endpoint was programmed with the date of recurrence as an eligible event or right censored at the date of the last medical record follow‐up. To evaluate recurrence‐free survival, the endpoint was programmed with the date of recurrence or date of death, whichever occurred first, as an eligible event; if neither of these events occurred, the outcome was right censored at the date of the last medical record follow‐up. To evaluate OS, the endpoint was programmed with the date of death as an eligible event or right censored at the date of the last medical record follow‐up.

## 3 RESULTS

Between 1 April 2015 and 31 December 2019, 618 patients with locoregional RCC underwent nephrectomy and were eligible for the study. Patient characteristics are presented in Table 1. In all, 136 (22%) patients were classified as modified high risk of recurrence.

**TABLE 1:** Demographic and disease characteristics of study population (*n* = 618), patients classified as high risk of recurrence after nephrectomy (*n* = 136), and patients classified as UISS high risk for recurrence (*n* = 52)

Characteristics, n (%)	All patients with an eligible nephrectomy	Patients classified as high risk of recurrence^a^	Patients classified as UISS high risk for recurrence^b^
All patients	618 (100)	136 (22)	52 (8.4)
Age at nephrectomy, year			
Median	62.87	67.03	67.54
Range, min, max	22.70, 88.99	35.84, 88.99	42.31, 84.67
**Sex**			
Female	200 (32.4)	40 (29.4)	16 (30.8)
Male	417 (67.5)	95 (69.9)	36 (69.2)
Other	1 (0.2)	1 (0.7)	0 (0)
Race documented	160 (25.9)	33 (24.3)	9 (17.3)
Asian	0 (0)	0 (0)	0 (0)
Black	57 (35.6)	13 (39.4)	2 (22.2)
White	103 (64.4)	20 (60.6)	7 (77.8)
Nephrectomy year			
2015	80 (12.9)	22 (16.2)	6 (11.5)
2016	131 (21.2)	34 (25.0)	15 (28.9)
2017	120 (19.4)	17 (12.5)	4 (7.7)
2018	128 (20.7)	29 (21.3)	14 (26.9)
2019	159 (25.7)	34 (25.0)	13 (25.0)
Nephrectomy type			
Full	295 (47.7)	125 (91.9)	49 (94.2)
Partial	323 (52.3)	11 (8.1)	3 (5.8)
ECOG PS documented	269 (43.5)	94 (69.1)	52 (100)
0	132 (49.1)	34 (36.2)	0 (0)
1	129 (48.0)	53 (56.4)	45 (86.5)
2	8 (3.0)	7 (7.5)	7 (13.5)
Tumor stage			
T1	420 (68.0)	2 (1.5)	0 (0)
T2	63 (10.2)	1 (0.7)	0 (0)
T3	135 (21.8)	133 (97.8)	52 (100)
T4	0 (0)	0 (0)	0 (0)
Nodal status			
N0	86 (13.9)	42 (30.9)	16 (30.8)
N1	10 (1.6)	10 (7.4)	7 (13.4)
NX	522 (84.5)	84 (61.8)	29 (55.8)
Tumor grade documented	595 (96.3)	134 (98.5)	52 (100)
1–2	472 (79.3)	68 (50.7)	21 (40.4)
3–4	123 (20.7)	66 (49.3)	31 (59.6)
TNM stage			
I	420 (68.0)	0 (0)	0 (0)
II	65 (10.5)	0 (0)	0 (0)
III	133 (21.5)	136 (100)	52 (100)
Clear cell predominance	457 (74.0)	114 (83.8)	43 (82.7)
Tumor necrosis present	156 (25.4)	69 (51.9)	33 (64.7)
Follow‐up plan determined at first post‐operative visit	552 (89.3)	128 (94.1)	51 (98.1)
Referral for discussion of AST	25 (4.5)	22 (17.2)	11 (21.6)
Surveillance	519 (94.0)	105 (82.0)	40 (78.4)
Not recorded/other	8 (1.5)	1 (0.8)	0 (0)

Abbreviations: AST, adjuvant systemic therapy; ECOG PS, Eastern Cooperative Oncology Group performance status; min, max, minimum, maximum; TNM, tumor, nodes, metastasis classification of malignant tumors.

^a^ Patients at modified high risk of recurrence are those that have a T stage of 3a or higher combined with a tumor grade of 2 or higher, regional lymph node metastasis, or both.

^b^ Patients at UISS high risk for recurrence are those that have a T stage of 3a or higher combined with a tumor grade of 2 or higher, regional lymph node metastasis, or both, as well as ECOG PS 1+.

The mean (SD) age at date of nephrectomy was 61.7 (11.2) years among all patients and 65.7 (10.7) years in the modified high risk of recurrence group. Forty‐eight percent of patients in the total population underwent full nephrectomy (vs. partial nephrectomy), whereas 92% of patients in the modified high risk of recurrence group underwent full nephrectomy (Table 1).

### 3.1 Post‐nephrectomy referrals and adjuvant therapy among patients at modified high risk of recurrence

Of the 136 patients with modified high risk of recurrence, 31 (23%) were referred to other providers for ongoing RCC‐related care (Table 2) and 25 (18%) with modified high risk of recurrence were referred to medical oncologists for discussion of AST options. Of the 25 referrals, 23 took place in 2018 or later. Three patients were intended to receive AST, at an average of 92 days after nephrectomy. One patient was treated with on‐label sunitinib and two patients participated in clinical trials of adjuvant immunotherapy.

**TABLE 2:** Referrals over time for patients at high risk of recurrence (*n* = 136)

Characteristic	Overall	2015	2016	2017	2018	2019
Total patients, *n* (%)	136 (100)	22 (16.2)	34 (25)	17 (12.5)	29 (21.3)	34 (25)
Follow‐up time, mean, years (SD)	1.72 (1.32)	2.50 (1.94)	2.55 (1.14)	2.02 (0.9)	1.26 (0.58)	0.62 (0.38)
Patients referred for discussion of AST, *n* (%)	25 (18.3)	1 (4.5)	1 (2.9)	0 (0)	7 (24.1)	16 (47.1)
Time to referral for discussion of AST, mean, days (SD)	55.6 (29.3)	86 (NE)	30 (NE)	—	55.7 (30.4)	55.2 (30.3)

Abbreviations: AST, adjuvant systemic therapy; NE, not evaluable; SD, standard deviation.

**FIGURE 1:**
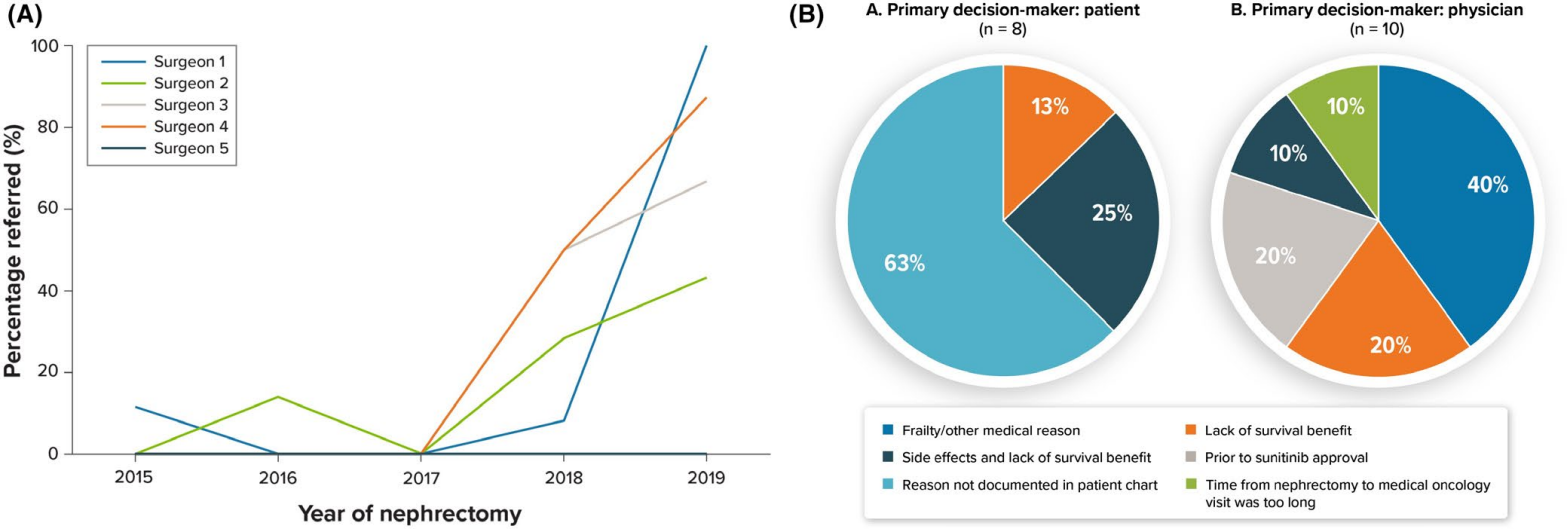
(A) Percentage of patients with high risk of recurrence referred for discussion of AST by individual surgeons. (B) Primary reason sunitinib was not received following referral for discussion of AST among patients with high risk of recurrence. The decision not to receive AST was primarily made by the oncologist in 10 of 22 (46%), by the patient in 8 (36%), and unrecorded in 4 (18%) cases based on clinic notes. AST, adjuvant systemic therapy

All referrals to medical oncology for discussion of AST were initiated by the urologist who performed the patient's nephrectomy. Although the overall percentage of patients referred for discussion of AST increased after 2017, individual surgeons referred varying percentages of patients at modified high risk of recurrence, ranging from 0% to 11% in 2016 to 0% to 100% in 2019 (Figure 1A).

In all, 88% of patients referred to medical oncologists for discussion of AST did not receive AST or enroll in a clinical trial. Among cases in which the patient was recorded as the primary decision‐maker not to receive adjuvant sunitinib, the reason for this decision was documented for 38% of patients, all of whom cited lack of survival benefit as the primary reason, with 25% reporting both lack of survival benefit and side effects (Figure 1B). Among cases in which the physician was documented as the primary decision‐maker, the recorded reasons varied: 40% cited frailty or other medical reasons, 20% cited lack of survival benefit, 20% cited no approved agent at the time (all prior to sunitinib approval), 10% cited the combination of side effects and lack of survival benefit, and 10% cited the time from nephrectomy to medical oncology visit being too long (Figure 1B).

### 3.2 Recurrence‐free survival and overall survival

Among patients with known recurrence status (*n* = 107), 39% of the patients developed recurrent RCC. The mean (SD) time to recurrence was 9.2 (9.1) months. Approximately 13% of patients died, and a mean (SD) time to death was 17.3 (14.1) months.

**FIGURE 2:**
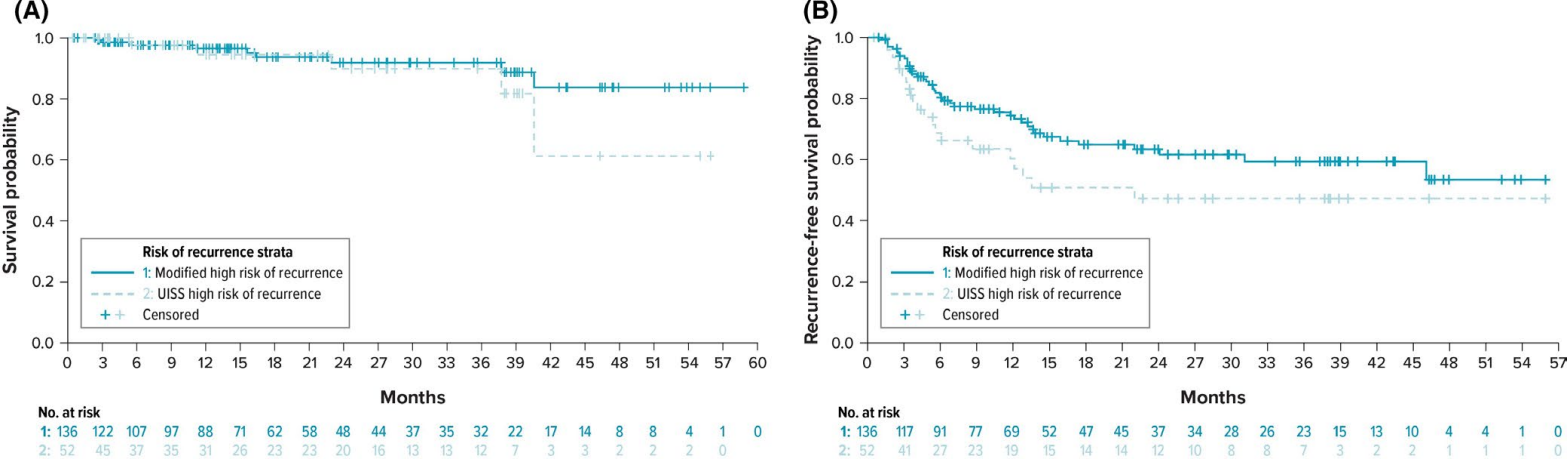
The Kaplan–Meier estimated (A) the overall survival and (B) recurrence‐free survival in patients at modified high risk of recurrence (*n* = 136) and the subset of these patients for which UISS risk was evaluable and high risk (*n* = 52). Median overall survival was not estimable and median time to recurrence was estimable only for the UISS high‐risk subgroup (22.0 months). UISS, UCLA Integrated Staging System

Eighty percent of patients classified as modified high risk of recurrence with performance status known were also considered high risk based on UISS (Table 1). Figure 2A shows the Kaplan–Meier estimated OS for the modified high risk of recurrence group (*n* = 136) and the subset of this group for which performance status was available and classified as high risk by UISS (*n *= 52). More than 90% of patients were censored (i.e., did not have an observable death event or had an unknown date of death), therefore median OS time was not estimable. Figure 2B shows the Kaplan–Meier curve for recurrence‐free survival for these two groups. Seventy‐one percent of patients were censored (did not have an observed recurrence event or had an unknown date of recurrence); therefore, median recurrence‐free survival was not estimable.

## 4 DISCUSSION

This is the first report, to our knowledge, of post‐nephrectomy referral patterns and real‐world use of AST in patients with locoregional RCC at high risk of recurrence. Among patients at modified high risk of recurrence, the rate of referral to medical oncologists for discussion of AST and clinical trials increased over time; however, referrals remained low (47%) as recently as 2019. Despite increasing referrals to medical oncologists, only three patients in this study received AST, either with on‐label sunitinib or in clinical trials.

We utilized a modified version of the UISS for RCC, namely removing ECOG PS as this was not available for 66% of patients in the study population, to classify patients as modified high risk of recurrence. Although similar to UISS, our modified high‐risk group likely included some patients who would be classified as moderate risk with UISS if performance status was available. Specifically, patients with tumors that were T3 and grade ≥2 would be classified as UISS high risk only if ECOG PS was greater than 0. The importance of performance status in this risk stratification highlights the need for routine assessment and recording of performance status at oncology visits, which is included in the American Society of Clinical Oncology Quality Oncology Practice Initiative (QOPI) Certification Program Standards.^16^ In prior trials of adjuvant therapy, risk stratification systems and inclusion criteria have varied. In the S‐TRAC trial, the most restrictive in requiring a high‐risk population by stage and the trial on which sunitinib's adjuvant approval is based, inclusion criteria included patients with tumor stage 3 or higher, regional lymph node metastasis, or both, on the basis of UISS criteria.^8^ Contrastingly, in the KEYNOTE‐564 trial of adjuvant pembrolizumab, additional patients with tumor stage 2 with nuclear grade 4 or sarcomatoid differentiation, tumor stage 3 with nuclear grade 1, and M1 disease with NED were included. These differences in inclusion criteria may contribute to small differences between study populations, however, there is considerable overlap across studies in defining patients at high risk of recurrence.^14^

Between the years 2017 and 2019, there was an upward trend in the percentage of patients with modified high risk of recurrence who were referred to a medical oncologist for discussion of AST. Although only two patients had such referrals from 2015 to 2017, 7 of 29 (24%) patients in 2018 and 16 of 34 (47%) patients in 2019 were referred to medical oncologists post‐operatively. This increase is temporally related to the approval of sunitinib as the first FDA‐approved adjuvant therapy for RCC in November 2017.^17^ Additionally, in 2017, a trial of adjuvant pembrolizumab (NCT03142334) opened at Duke University (Durham, NC, USA),^18^ which may have contributed to increased referrals to medical oncology for discussion of adjuvant options. Still, as recently as 2019, more than half of patients at modified high risk of recurrence were not referred to medical oncologists post‐operatively.

At most three patients received AST––one patient received on‐label sunitinib and two patients participated in a clinical trial of adjuvant immunotherapy. We attempted to capture the reasons behind the decision not to prescribe AST that was documented in the medical records of the 22 patients who saw medical oncologists but did not receive AST. Noting methodology was limited to retrospective medical record review, whether the primary decision‐maker was the physician or the patient, commonly cited reasons that patients did not receive sunitinib were lack of survival benefit and associated side effects. Physicians cited patient co‐morbidities or frailty as another reason against the use of adjuvant sunitinib.

The mean time from nephrectomy to referral for discussion of AST was 55.6 days (SD 29.3) in our study. In S‐TRAC, sunitinib was initiated within 3–12 weeks after nephrectomy and in KEYNOTE‐564, patients were randomized within 12 weeks after nephrectomy.^8^, ^14^ Additional ongoing trials of immunotherapy agents in high‐risk RCC (NCT03138512, NCT03288532, and NCT03024996) require randomization within 12 weeks after nephrectomy.^18^, ^19^, ^20^, ^21^ Prompt referral to medical oncologists after nephrectomy is necessary in order to ensure sufficient time for risk–benefit discussions and assessment of eligibility for adjuvant clinical trials.

Prior to our study, it has remained unclear what uptake of adjuvant therapy and trials in high‐risk locoregional RCC has been and whether patients are being referred for consideration of these therapies in real‐world practice. The current study is a review of patients treated at a single, large academic medical center, which may limit generalization across treatment settings. Despite increasing rates of referral for discussion of AST, these rates remained below 50% and subsequent receipt of AST remained low in patients with RCC at high risk of recurrence. The use of adjuvant sunitinib in RCC remains controversial given the conflicting results of published trials and lack of demonstrated survival benefit; however, it is an FDA‐approved therapy in this setting and discussion of AST is a category 2B recommendation in the 2021 NCCN Guidelines.^11^, ^22^, ^23^, ^24^ Additionally, trials of adjuvant immunotherapy are ongoing and initial results are promising with improved disease‐free survival with adjuvant pembrolizumab, potentially presenting future options for which may present additional options for patients to consider. These findings highlight the need for continued efforts at identifying effective AST and referring patients that may benefit from these interventions.

## CONFLICT OF INTEREST

Elizabeth Esterberg and LaStella Miles are employees of RTI Health Solutions who were paid consultants to Pfizer in connection with the development of this manuscript. Giovanni Zanotti and Azah Borham are employees of Pfizer. Michael R. Harrison receives research funding from Pfizer. Hannah Dzimitrowicz has no disclosures.

## ETHICAL APPROVAL

This study was approved by the institutional review board and waivers for obtaining informed consent were granted.

## Data Availability

The data that support the findings of this study are available from the corresponding author upon reasonable request.
